# An Audit of Indications, Complications, and Justification of Hysterectomies at a Teaching Hospital in India

**DOI:** 10.1155/2014/279273

**Published:** 2014-01-02

**Authors:** Deeksha Pandey, Kriti Sehgal, Aashish Saxena, Shripad Hebbar, Jayaram Nambiar, Rajeshwari G. Bhat

**Affiliations:** ^1^Department of OBGYN, KMC Manipal, Manipal University, 4/1 KMC Flats, KMC Campus, Manipal 576104, Karnataka, India; ^2^KMC Manipal, Manipal University, India

## Abstract

*Objective*. Aim of this audit was to analyze indications, complications, and correlation of preoperative diagnosis with final histopathology report of all hysterectomies, performed in a premier teaching hospital. *Methods*. Present study involved all patients who underwent hysterectomy at a premier university hospital in Southern India, in one year (from 1 January, 2012, to 31 December, 2012). *Results*. Most common surgical approach was abdominal (74.7%), followed by vaginal (17.8%), and laparoscopic (6.6%) hysterectomy. Most common indication for hysterectomy was symptomatic fibroid uterus (39.9%), followed by uterovaginal prolapse (16.3%). Overall complication rate was 8.5%. Around 84% had the same pathology as suspected preoperatively. Only 6 (5 with preoperative diagnosis of abnormal uterine bleeding and one with high grade premalignant cervical lesion) had no significant pathology in their hysterectomy specimen. *Conclusion*. Hysterectomy is used commonly to improve the quality of life; however at times it is a lifesaving procedure. As any surgical procedure is associated with a risk of complications, the indication should be carefully evaluated. With the emergence of many conservative approaches to deal with benign gynecological conditions, it is prudent to discuss available options with the patient before taking a direct decision of surgically removing her uterus.

## 1. Introduction

Hysterectomy is the second most frequently performed major surgical procedures on women all over the world, next only to cesarean. In US, approximately 600,000 hysterectomies are performed each year [1]. In India no national statistics for hysterectomy is available. A study conducted in a northern state of India (Haryana) states that incidence of hysterectomy was 7% among married women above 15 years of age [2]. Another study from a western state (Gujarat) pointed out that 7-8% of rural women and 5% of urban women had already undergone hysterectomy at an average age of 37 years [3].

Indications of hysterectomy vary from benign condition to malignancies of genital tract. Term “hysterectomy” though means removal of uterus, in practice it has a much wider classification depending upon the indication. At times it is done without removal of the cervix (supracervical hysterectomy) or with removal of adnexa (hysterectomy with salpingo-oophorectomy). It can also be a part of staging laparotomy or radical hysterectomy. Hysterectomy can be performed abdominally, vaginally or through abdominal ports with help of a laparoscope. Approach depends on surgeon's preference, indication for surgery, nature of disease, and patient characteristics.

As any other surgery, hysterectomy is also associated with intraoperative and postoperative complications. Rates of various complications with hysterectomy have been reported in the range from 0.5% to 43% [4].

Emergence of effective medical and conservative treatment for benign conditions in the uterus is now posing a question mark regarding the justification of hysterectomy. It has been realized that uterus should not be considered as a vestigial organ after child bearing. Studies have proved that following hysterectomy women suffer with bothersome psychosexual functions [5] and increased incidence of vaginal prolapse due to deficiency of supporting ligaments. It has also been hypothesized that ovarian endocrinal function weans of more rapidly after removal of their target organ. Mean age of onset of menopause in those who underwent hysterectomy is 3.7 years earlier than average, even when the ovaries are preserved [6].

Aim of this audit was to analyze indications, complications, and correlation of preoperative diagnosis with final histopathology report of all hysterectomies, performed in a premier teaching hospital. As there are no hysterectomy audits published from India recently, present study may provide a basis for a future audit of our gynecologic practice and for comparison of our practice with others.

## 2. Material and Methods

Present study involved all patients who underwent hysterectomy at Kasturba Hospital (KH), a teaching hospital affiliated with Kasturba Medical College (KMC), Manipal, Manipal University in Southern India, in a span of one year (from 1 January, 2012, to 31 December, 2012). The study was approved by institutional ethical review board. There were no exclusion criteria. Patients were identified by medical record tracking using ICD-9 codes. Case records then were reviewed to collect patient characteristics, indication for surgery, approach, complications, and length of hospital stay. Intensive care admissions and repeat laparotomies were also assessed.

All elective as well as emergency hysterectomies (including obstetric hysterectomies) were analyzed. Cases where intraoperative consultation was sought by other specialties and where on table decision to proceed with hysterectomy was taken were also included. Abdominal hysterectomies included supracervical hysterectomy, total hysterectomy (TAH), and hysterectomy with unilateral (TAH with USO) or bilateral salpingo-ovariotomy/oophorectomy (TAH with BSO). It also included hysterectomy done as a part of staging laparotomy for ovarian tumor and radical hysterectomy done for early stage cervical carcinoma. Vaginal hysterectomy included vaginal hysterectomy with pelvic floor repair (VH with PFR) for uterovaginal prolapse and also nondescent vaginal hysterectomy (NDVH) for indications other than uterovaginal prolapse. Laparoscopic hysterectomy group had both laparoscopic assisted vaginal hysterectomy (LAVH) and total laparoscopic hysterectomy (TLH).

Among all cases various indications were reviewed. Some of the women had more than one indication. As a part of subanalysis, indications where hysterectomy was performed in less than 30 years of age were carefully reviewed. Intraoperative blood loss, injury to vital structures, and conversion of planned route were compared among various approaches.

At the end main postoperative histopathology diagnosis was recorded. Preoperative indication was compared with pathologist's report after surgery. Hysterectomy was considered justified if the pathology report verified the indication for surgery or showed a significant alternative pathology.

All calculations were made using SPSS 21.0 IBM Statistics released August 2012 (IBM Corporation 1, New Orchard Road, Armonk, NY, 10504-1722 USA). Categorical variables were compared using the Fisher exact test (2 sided). Significance was set at *P* < 0.05.

## 3. Results

In the year 2012 in our hospital a total of 45,423 women attended our outpatient department (OPD) for gynecological complaints and 5,837 were admitted in the hospital. Out of which 859 patients were treated with some major surgical procedure which included 527 hysterectomies. Thus 9% of total gynecological admissions ended up with hysterectomy. Around 60% of major gynecological surgeries were hysterectomy. Records were obtained and information was analyzed. None of the cases were excluded from final analysis.

**Figure 1:**
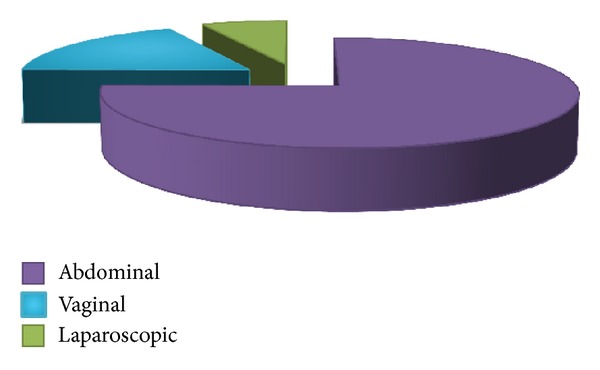
Proportion of various approaches of hysterectomy.

**Figure 2:**
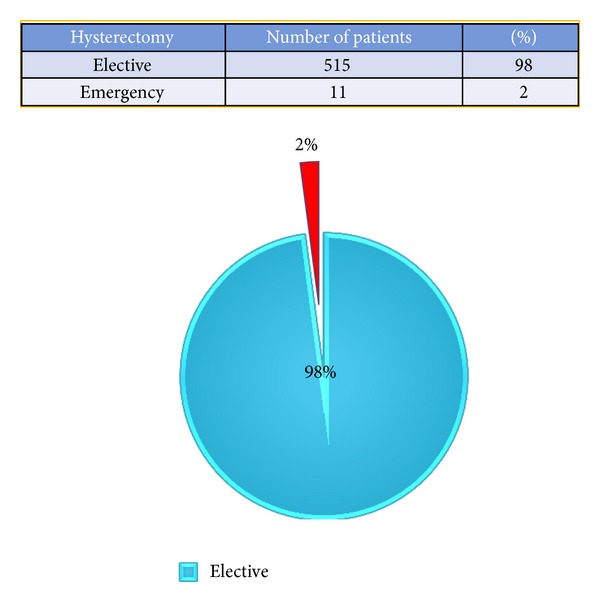
Elective versus emergency (including obstetric hysterectomies) hysterectomies.

Most common surgical approach was abdominal (*n* = 394 [74.7%]), followed by vaginal (*n* = 94 [17.8%]), and laparoscopic (*n* = 35 [6.6%]) (Figure 1). Most of the hysterectomies (98%) were done as elective surgical procedure (Figure 2). Mean age of women undergoing hysterectomy was 48 ± 9.9 years (abdominal: 46.2 ± 9.0, vaginal: 56.2 ± 10.5, laparoscopic: 46.7 ± 5.6 years). Ten women who were less than or equal to 30 years underwent abdominal hysterectomy (Table 1). Four of these were for obstetric indication (one postpartum hemorrhage (PPH), one ruptured uterus, one puerperal sepsis not responding to medical management), and one had ruptured her uterus during a road traffic accident (RTA). Three had malignant ovarian tumor. One endometriosis refractory to medical management and one fibroid uterus with severe dysmenorrhea insisted on undergoing hysterectomy. Among the 113 patients who underwent hysterectomies in the age group of 30 to 40 years (Table 1), all of them had completed their families. With all of these women feasible nonsurgical or conservative surgical options were discussed and/or tried.

Most common indication for hysterectomy was symptomatic fibroid uterus (*n* = 210 [39.9%]), followed by utero-vaginal prolapse (*n* = 86 [16.3%]). Other indications being dysfunctional uterine bleeding (DUB) (*n* = 43 [8.1%]), adenomyosis (*n* = 21 [3.9%]), endometriosis (*n* = 7 [1.3%]), benign (*n* = 42 [7.9%]) and malignant (*n* = 47 [8.9%]) ovarian tumors, endometrial hyperplasia (*n* = 25 [4.7%]) and endometrial cancer (*n* = 20 [3.7%]), premalignant disease of cervix (*n* = 17 [3.2%]), and early stage cervical cancer (*n* = 4 [0.7%]). Less common indications were recurrent postmenopausal bleeding of undetermined cause (*n* = 16 [2.8%]) and chronic pelvic inflammatory disease (PID) (*n* = 8 [1.5%]). Obstetric hysterectomy was performed in 8 (1.5%) cases (Tables 2 and 3).

Two women undergoing TAH with BSO sustained bowel injury, one in a case of tubo-ovarian mass and other in a malignant ovarian tumor. Bladder was injured accidentally in four cases of abdominal hysterectomies (1.01%) and in one VH with PFR (1.06%). One patient during abdominal hysterectomy had ureteric injury which required Boari's flap. Postoperatively ten (2.5% of abdominal approach) patients had wound infection or gaping. Two (0.5%) had burst abdomen. Two patients (also belonging to abdominal approach) had pelvic abscess which were managed conservatively. Two women had postoperative intestinal obstruction. One was managed conservatively. Other underwent exploratory laparotomy, adhesiolysis with resection anastomosis on the twelfth day following hysterectomy. On the sixth postoperative day following laparotomy she succumbed due to respiratory distress syndrome (Table 4).

Twenty-one women (3.9%) had blood loss of more than 1000 mL during surgery and required multiple transfusions. Among these twenty had undergone abdominal hysterectomy (three PPH, two ruptured uterus, two puerperal sepsis, six fibroids, two benign and three malignant ovarian tumor, two endometrial cancer, and one cervical cancer) and one underwent NDVH for DUB.

One VH had to be converted to laparotomy due to adhesions. For one VH minilaparotomy was done to achieve complete hemostasis. One staging laparotomy for malignant ovarian tumor was abandoned because of frozen pelvis, and only biopsies were taken in order to start neoadjuvant chemotherapy.

Five women required intensive care admission after surgery. One lady died after two days following peripartum hysterectomy. One lady developed multiple organ dysfunction syndrome (MODS) and was discharged at request in a moribund condition. Mean duration of hospital stay in abdominal, vaginal, and laparoscopic approach was, respectively, 8.7 ± 2.8, 6.8 ± 1.6, and 6.1 ± 2.6 days.

In a total of 47 [8.9%] cases intraoperative consultation was sought by either an oncosurgeon, urosurgeon, or a general surgeon. Most of these cases were abdominal hysterectomies, where in 15 cases of malignant disease an oncosurgeon was part of the team. In 26 cases assistance from general surgeons was sought for release of dense bowel adhesions. Assistance from urosurgeons was sought in six cases.

For correlation of final histopathology with preoperative diagnosis, 11 emergency hysterectomies done to save life were excluded. Among the rest 516 hysterectomies 434 (84.1%) had the same pathology as it was suspected preoperatively. Around a fifth (101, 19.6%) was found to have an extra pathology that might have required surgical treatment. 85 (16.4%) were found to have a pathology which was different from the preoperative diagnosis. Among these were six patients with a preoperative diagnosis of malignant ovarian tumor, out of which three came out to be benign, one was Krukenberg's tumor, one was GIST, and one turned out to be fallopian tube carcinoma. One case preoperatively diagnosed as hydrosalpinx also turned out to be fallopian tube carcinoma. Three patients with preoperative normal Pap smears had cervical intraepithelial neoplasia: high grade (CIN II/III) in the hysterectomy specimen. Two cases of prolapse had incidental finding of squamous cell carcinoma in histopathology. Two cases operated for endometrial hyperplasia had foci of carcinoma in our series. Only six (five with preoperative diagnosis of AUB and one with CIN II) had no significant pathology in their hysterectomy specimen.

## 4. Discussion

In our institution in a span of one year we performed 527 hysterectomies. Most of these were abdominal (75.5%) followed by vaginal (17.8%) and laparoscopic (6.6%). Almost the same observations come from Canada (abdominal 78%, vaginal 14%, and laparoscopic 5.9%) [7]. In Hong Kong the proportion of laparoscopic seemed a little higher (abdominal 70.2%, vaginal 15.9%, and laparoscopic 13.8%) [8]. A decade long data from UK shows the same trend of abdominal hysterectomies being five- to sixfold more common than vaginal approach [9].

Most common indication in abdominal approach was fibroid uterus (total 39.9% and out of abdominal 52.7%), while in vaginal it was uterovaginal prolapse (total 16.3% and out of vaginal 91.5%). A study evaluating the appropriateness of recommendation for hysterectomy in USA also found fibroid (60%) to be the most common indication followed by prolapse (11%) [10]. In the data from Hong Kong, fibroids constituted even higher proportion of indications for abdominal (73.7%) and genital prolapse was the most common indication (96.2%) for vaginal hysterectomy [8]. In Pakistan also the most common reason for hysterectomy was fibroid (33%) followed by uterovaginal prolapse (19%) and DUB in 18 (12%) [11]. In a recent study from Africa too uterine fibroids were the most common reason of performing hysterectomy (23%); however there it was followed by DUB (14.9%) [12]. In a study from Canada, however, the commonest indication of hysterectomy was DUB (26.4%), followed by fibroid uterus (16.0%) [7].

Overall complication rate was 8.5%. Abdominal approach had a complication rate of 10.9%, as compared to 2.1% in vaginal approach (*P* < 0.05). In Hong Kong the incidence of complications for vaginal hysterectomy (17.0%) was lower than that for both abdominal (26.4%) (*P* < 0.001) and laparoscopic hysterectomy (23.9%) (*P* < 0.05) [8]. Like this in many more studies it has been stated that vaginal hysterectomy has a lower incidence of complications. Cochrane review concluded that vaginal hysterectomy should be performed in preference to abdominal hysterectomy, where possible. Where vaginal hysterectomy is not possible, a laparoscopic approach may avoid the need for an abdominal hysterectomy [13]. However in our opinion data should be analyzed carefully keeping in mind that hysterectomies to save life (for obstetric and malignant conditions) are usually done through the abdominal approach. These hysterectomies for an obvious reason are more prone to have higher incidence of complications. On comparing the complication rate among surgeries performed for malignant and obstetric versus benign disease, the complication rate was not significant (*P* < 0.05).

Mean duration of stay in our hospital following hysterectomy by any route was more than other studies. In abdominal, vaginal and laparoscopic approach was, respectively, 8.7 ± 2.8, 6.8 ± 1.6, and 6.1 ± 2.6 days, as compared to study from Hong Kong where it was found to be 6.7 ± 2.5, 4.9 ± 2.4, and 4.2 ± 3.4 days [8].

Hysterectomy remains a matter of diverse debate owing to its physical, emotional, economic, sexual, and medical significance to women [14]. In a study to judge appropriateness of nonemergency and nononcologic hysterectomies in USA, indications were often found to be inappropriate [10]. Hysterectomy was justified in 98.9% women in our series based on postoperative histopathology report of the specimen. However we hypothesize that 43 cases of DUB, 17 cases of CIN, and 16 cases of postmenopausal bleeding without any significant pathology could have been managed conservatively. Nine cases of obstetric hysterectomies could have been avoided by disseminating awareness among peripheral centers for early referral of high risk obstetric cases and by attempting a rather conservative approach of uterine artery embolization (UAE), as the expertise and facilities are available in our center.

There is a concern among the medical and health policy community that hysterectomy is used too frequently as a first line treatment. It is a well-known fact that in order to achieve the most favorable outcome, appropriateness of surgery must be carefully evaluated along with all available options in context of the disease process like medical management, endometrial ablation, UAE, and levonorgestrol intrauterine system (LNG-IUS). Oral medication suits a minority of women long term [15]. Endometrial resection and ablation offers an alternative to hysterectomy for heavy menstrual bleeding. Although hysterectomy is associated with a longer operating time, a longer recovery period, and higher rates of postoperative complications, it offers permanent relief. The initial cost of endometrial destruction is significantly lower than hysterectomy, but, since retreatment is often necessary, the cost difference narrows over time [16]. Hysterectomy, reduces menstrual bleeding more than medical treatments at one year but LNG-IUS may be comparable in improving quality of life. Evidence for longer-term comparisons however is weak and inconsistent [15]. UAE appears to have an overall patient satisfaction rate similar to hysterectomy and myomectomy while offering an advantage with regards to a shorter hospital stay and a quicker return to routine activities. However, it is associated with higher rate of minor complications and increased likelihood of requiring surgical intervention within 2–5 years of the initial procedure [17].

According to Magon et al. hysterectomy is a surgery which has been used and misused, underused, and abused at different times in gynecology [18]. Hysterectomy is used commonly to improve the quality of life; however at sometimes it is a lifesaving procedure. As any surgical procedure is associated with a risk of complications, the indication should be carefully evaluated. With the emergence of many conservative approaches to deal with benign gynecological conditions, it is prudent to discuss available options with patient before taking a direct decision of sacrificing her uterus.

## Figures and Tables

**Table 1:** Approach of hysterectomy in relation to age group of the women.

Age group	Abdominal (75.5%) *n* = 396 (+2)					Vaginal (17.8%) *n* = 94		Laparoscopic (6.6%) *n* = 35	
	TAH *n* (%)	TAH + USO *n* (%)	TAH + BSO *n* (%)	Staging laparotomy *n* (%)	Radical hysterectomy *n* (%)	VH + PFR *n* (%)	NDVH *n* (%)	LAVH *n* (%)	TLH *n* (%)
21–30 (*n* = 10)	05 (0.9)	01 (0.2)	01 (0.2)	03 (0.6)	00	00	00	00	00
31–40 (*n* = 113*)	47 (8.9)	08 (1.5)	32 (6.1)	12 (2.3)	00	05 (0.9)	04 (0.7)	04 (0.7)	00
41–50 (*n* = 239**)	22 (4.2)	08 (1.5)	147 (27.9)	18 (1.5)	02 (0.4)	11 (2.1)	05 (0.9)	24 (4.5)	01 (0.2)
51–60 (*n* = 100)	01 (0.2)	00	38 (7.2)	21 (3.9)	02 (0.4)	32 (6.1)	01 (0.2)	05 (0.9)	00
61–70 (*n* = 54)	00	00	13 (2.5)	06 (1.1)	01 (0.2)	33 (6.3)	00	01 (0.2)	00
71–80 (*n* = 9)	00	00	02 (0.4)	04 (0.7)	00	03 (0.6)	00	00	00
81–90 (*n* = 2)	00	00	02 (0.4)	00	00	00	00	00	00

*One underwent supracervical hysterectomy with USO. **One LAVH converted to TAH.

**Table 2:** Indication of hysterectomy (an overall view).

Indications	Number of patients (*n*)	Percentage (%)
Fibroid	210	39.8%
Prolapse	86	16.3%
DUB	43	08.1%
Adenomyosis	21	03.9%
Endometriosis	07	01.3%
Ovarian tumor (benign)	42	07.9%
Ovarian tumor (malignant)	47	08.9%
Endometrial hyperplasia	25	04.7%
Endometrial carcinoma	20	03.7%
CIN	17	03.2%
Cervical cancer	04	00.7%
Postmenopausal bleeding	16	02.8%
Chronic PID	07	01.3%
PPH	04	00.7%
Puerperal sepsis	01	00.1%
Ruptured uterus	04	00.7%

*Some women had more than one indication.

**Table 3:** Indication of hysterectomy in relation to the approach.

Indications	Abdominal					Vaginal		Laparoscopic	
	TAH	TAH + USO	TAH + BSO	Staging laparotomy	Radical hysterectomy	VH + PFR	NDVH	LAVH	TLH
Fibroid (210)	54 (10.2)	12 (2.3)	124 (23.5)	00	00	02 (0.4)	05 (0.9)	12 (2.3)	01 (0.2)
Prolapse (86)	00	00	01 (0.2)	00	00	83 (15.7)	00	02 (0.4)	00
DUB (43)	08 (1.5)	00	26 (4.9)	00	00	00	03 (0.6)	06 (1.1)	00
Adenomyosis (21)	01 (0.2)	01 (0.2)	15 (2.9)	00	00	00	01 (0.2)	03 (0.6)	0 0
Endometriosis (07)	00	01 (0.2)	06 (1.1)	00	00	00	00	00	00
Ovarian tumor benign (42)	00	04 (0.8)	35 (6.6)	00	00	00	00	02 (0.2)	00
Ovarian tumor malignant (47)	00	00	02 (0.4)	45 (8.5)	00	00	00	00	00
Endometrial hyperplasia (25)	01 (0.2)	00	18 (3.4)	00	00	00	02 (0.4)	04 (0.8)	00
Endometrial cancer (20)	00	00	00	19 (3.6)	01 (0.2)	00	00	00	00
CIN (17)	03 (0.6)	00	09 (1.7)	00	01 (0.2)	01 (0.2)	00	03 (0.6)	00
Cervical cancer (04)	00	00	00	00	03 (0.6)	01 (0.2)	00	00	00
Postmenopausal bleeding (16)	00	00	13 (2.5)	00	00	00	00	03 (0.6)	00
Chronic PID (07)	01 (0.2)	02 (0.4)	03 (0.6)	00	00	00	00	01 (0.2)	00
PPH	04 (0.7)	00	0	00	00	00	00	00	00
Puerperal sepsis	00	01 (0.2)	00	00	00	00	00	00	00
Ruptured uterus	03 (0.6)	00	00	00	00	00	00	00	00

**Table 4:** Complications of hysterectomy in relation to the approach.

Complications	Abdominal (43, 10.9%)					Vaginal (2, 2.1%)	
	TAH	TAH + USO	TAH + BSO	Staging laparotomy	Radical hysterectomy	VH + PFR	NDVH
Intraoperative complications							
Bowel injury (02)	00	00	02	00	00	00	00
Bladder injury (05)	01	00	03	00	00	01	00
Ureteric injury (01)	01	00	00	00	00	00	00
Blood loss (>1000 mL) (21)	07	01	06	05	01	00	01

Postoperative							
Wound infection/gape (10)	01	00	06	03	00	00	00
Burst abdomen (2)	0	0	2	0	00	00	00
Pelvic abscess (2)	0	0	2	0	00	00	00
Intestinal obstruction (2)	1	0	1	0	00	00	00
